# Attitudes toward palliative care among cancer patients: a multi-method study

**DOI:** 10.3389/fpubh.2025.1511697

**Published:** 2025-03-05

**Authors:** Meiying Zhang, Yuxia Zhao, Yifu Lu, Mengyun Peng

**Affiliations:** ^1^Department of Diagnostic Imaging, National Cancer Center/National Clinical Research Center for Cancer/Cancer Hospital, Chinese Academy of Medical Sciences and Peking Union Medical College, Beijing, China; ^2^Shantou University Medical College, Shantou University, Shantou, China; ^3^School of Nursing, Suzhou Medical College, Soochow University, Suzhou, China

**Keywords:** palliative care, attitudes, cancer, quantitative study, qualitative study

## Abstract

**Background:**

Palliative care plays a crucial role in improving the quality of life for cancer patients, particularly those in advanced stages of the disease. Despite its proven benefits, attitudes toward palliative care vary widely among patients due to cultural beliefs, personal values, and awareness of available services. Understanding cancer patients’ perspectives on palliative care is essential for enhancing end-of-life care strategies and ensuring that interventions align with their preferences. However, limited research has explored patients’ attitudes toward palliative care in China, highlighting the need for further investigation.

**Objectives:**

To explore the current status of cancer patients’ palliative care attitudes, identify subgroups of attitudes and examine influencing factors for different subgroups; and understand the cancer patients’ perceptions of palliative care.

**Methods:**

A multi-method design was used. 541 cancer patients participated from March to June 2024. A latent profile analysis (LPA) was conducted to identify subgroups. The differences between the variables including sociodemographic characteristics and subgroups were explored, and participants also responded to open-ended questions about what perceptions on palliative care, and content analysis identified themes most frequently reported.

**Results:**

Palliative care attitudes among cancer patients were low. Four different subgroups of palliative care attitudes and three themes about perspectives were confirmed. Education status, occupational status, primary caregivers, type of insurance, cancer stage, anxiety, and level of palliative care knowledge were significant factors affecting different groups (*p* < 0.05).

**Conclusion:**

Majority of cancer patients had poor attitudes toward palliative care, confirming the major factors and perspectives of palliative care. These results emphasize the importance that should be given to the dissemination of knowledge and education about palliative care for cancer patients, and to improve the acceptance and recognition in order to promote palliative care practice.

## Introduction

In recent decades, there has been growing attention to palliative care in healthcare systems around the world. Palliative care (PC) is an approach that improves the quality of life for patients with life-threatening illnesses and their families by preventing and alleviating suffering through early identification, accurate assessment, and pain management (1). Although there is evidence to suggest that cancer patients receiving palliative care can reduce pain and depressive symptoms and improve their quality of life (2–4), the early referral rate for palliative care remains low, and there are many challenges associated with palliative care implementation (5, 6).

## Background

Cancer is one of the world’s leading causes of death. Global Cancer Statistics 2020 showed that China accounted for 24% of newly diagnosed cancer cases and 30% of cancer-related deaths worldwide in that year. China’s age-standardized incidence and mortality rates are higher than the global average, and the burden of cancer is continues to rise (7). However, 2021 Quality of Death Index showed that China ranks low in the world (53/81). Therefore, there is an urgent need to develop strategies and models to improve the quality of end-of-life care and death for cancer patients (8).

Palliative care is a form of active, holistic care for all people who are experiencing health-related suffering due to critical illnesses, especially those who are terminally ill patients. Its goal is to enhance the quality of life for both patients and their families (9). As an integral part of standard oncology practice, numerous studies have confirmed that cancer patients would benefit from palliative care (5, 6, 10). However, palliative care practices face a range of obstacles. Shalev et al. (10) found that low levels of palliative care awareness among cancer patients negatively affected their willingness to seek it. Cancer patients’ misperceptions, such as confusing palliative care with stopping treatment and believing that receiving palliative care is equivalent to “death,” also contributing to underuse of palliative care (10, 11). Furthermore, several international studies have shown that cancer patients avoid end-of-life discussions and that topics related to death remain ‘taboo’ (12). These factors may influence cancer patients’ attitudes toward palliative care.

China is a country deeply influenced by traditional cultural beliefs. In traditional Chinese culture, talking about death is ‘taboo,’ and families who choosing palliative care for patients over active treatment are sometimes perceived as ‘unfilial.’ As a result, palliative care remains in its early stages of development in China (13–15). More than half of the African cancer patients had willingness to accept palliative care, the degree of willingness palliative care was high (16). Findings from America demonstrated that bladder cancer have highly accurate knowledge of palliative care services, attitudes and beliefs surrounding palliative care were overall positive (17). However, Collins et al. (18) conducted a qualitative interview in Australia, and demonstrated that patients viewed palliative care as a ‘secondary treatment’ and reported negatively that palliative care would diminish the achievement of other cure-focused treatments. A Belgian study also found cancer patients had negative attitudes toward starting a conversation about palliative care with healthcare provider (19). These findings suggest that attitudes toward palliative care for cancer patients in different cultural backgrounds show significant differences. However, the attitudes of Chinese cancer patients toward palliative care remain unclear.

Most previous studies have assessed patients’ palliative care attitudes using visual analog scales. However, relying solely on simplified categorical scoring may overlook individual-level differences across subgroups. As attitude represents a predispositional evaluation, neglecting these individual differences among subgroups could affect the effectiveness of intervention strategies. Given the inconsistencies and limitations of existing studies, a multi-method approach is necessary to further validate the current state of cancer patients’ attitudes toward palliative care and to achieve a more precise classification of these attitudes.

## Purpose

This study aimed at filling this gap, latent profile analysis (LPA) is an individual-centered approach that uses continuous explicit variables to cluster data so that group heterogeneity can be explored (20, 21). Therefore, this study aims to explore the current status of cancer patients’ palliative care attitudes, and identify subgroups of attitude and examine influencing factors for different subgroups using LPA; understand the cancer patients’ perceptions of palliative care using qualitative study. The quantitative research hypotheses and qualitative research question are as follows:

*H1*: There are distinct latent subgroups of cancer patients based on attitudes toward palliative care.

*H2*: Sociodemographic characteristics (e.g., age, gender) significantly influence subgroup in palliative care attitudes.

Q1: How do cancer patients perceive and understand palliative care?

## Methods

### Study design and participants

This multi-method study used the STROBE checklist. Quantitative data were collected using an online questionnaire to understand the level of palliative care attitudes in cancer patients. Qualitative data were collected and coded using open-ended survey questions to explain and validate the root causes of quantitative results. The narrative comments from open-ended questions are typically meant to provide a forum for explanations, meanings and new ideas, the purposes and functions of qualitative and quantitative data in this study on questionnaires are different, yet complementary (22, 23). A convenient sample of cancer patients was selected between March 30 and June 30 of 2024 from the outpatient setting of the Cancer Hospital Chinese Academy of Medical Sciences. The trial population consisted of cancer patients of any type and stage who agreed to participate. The inclusion criteria were: (a) patients aged 18 and older with a confirmed cancer diagnosis, and (b) no noticeable cognitive impairment. Patients who unable to fill out all questionnaires due to physical or emotional stress were excluded. Larger sample sizes in surveys are known to yield more accurate and representative results. According to MacCallum (24), the minimum required sample size of 100, or the sample size to variable ratio should be at least 5. Finally, 541 cancer patients participated and completed the study.

## Measures

### Sociodemographic characteristics

Sociodemographic characteristics divided into two main sections: sociodemographic information, including age, gender, marital status, ethnicity, religion, educational status, occupational status, residence, average family income per month, type of medical insurance, and primary caregivers; the disease-related information, including the duration of illness, cancer stage, level of pain, level of anxiety, level of depression, level of disease knowledge, level of palliative care knowledge, experiences of palliative care-related education or training.

### Palliative care attitudes scale (PCAS-9)

The Palliative Care Attitudes Scale (PCAS-9) was developed by Perry (25) in 2020 and translated by Liu (26) in 2022. It includes 9 items across 3 dimensions: emotional (3 items), cognitive (3 items), and behavioral (3 items). The emotional dimension was reverse scored on a 6-point scale ranging from 1 (not at all stressful) to 6 (extremely, extremely stressful). The cognitive and behavioral dimensions were rated on a 7-point scale ranging from 1 (definitely no) to 7 (definitely yes). The total score ranges from 9 to 60, with higher scores indicating more positive palliative care attitudes. The PCAS-9 in this study has a Cronbach’s alpha of 0.894.

### Qualitative question

To allow participants to express their attitudes toward palliative care in own words and to help us better understand their perspectives, we included an open-ended question: What is your perspective on palliative care? There was no word limit on responses.

### Data collection

This study was conducted on online, which is called “wenjuanxing,” the largest e-questionnaire platform in China. The online questionnaire was set up so that each IP address could only submit one response to prevent duplicate answers. Our research team contacted the directors of the nursing departments and after explaining the study’s purpose, process and obtaining consent, one of our team members sent the questionnaire URL link to the participants who met the inclusion criteria at the outpatient clinic. The first page of the questionnaire described the study in detail and provided informed consent. Only those who selected “Yes, I agree to join” were allowed to complete the rest of the questionnaire.

### Data analysis

Profiles were found using LPA in Mplus 8.3. LPA is widely applied to estimate the number of subpopulations in an example. LPA can employ hypothetical categorical variables to clarify the relationship between exogenous continuous-type indicators, allowing for the evaluation of the relationship between exogenous indicators while maintaining local independence among them. The log likelihood, Akaike Information Criterion (AIC), Bayesian Information Criterion (BIC), adjusted BIC, entropy, Lo–Mendell–Rubin, and bootstrapped likelihood ratio tests were employed to evaluate model fit and identify the optimal number of categories. To determine the best model, and the models from each category’s fitting results were mixed with the indicators.

The quantality data were analyzed using SPSS 26.0. For categorical data, we utilized frequency and composition ratios; for continuous data, we used mean and standard deviation. The χ^2^ test was used to analyze categorical variables between groups. The analysis of variance (ANOVA) was performed for assessing continuous variables across groups. A multivariate logistic regression model was used to investigate socioeconomic variances. *p* < 0.05 indicates a statistically significant difference.

The qualitative data from open-ended questions were evaluated utilizing content analysis methods to interpret the text data through a coding process, followed by theme identification (27). This approach enables immersion in the data, leading to new insights and category generalization. It is particularly beneficial for analyzing large volumes of textual data (28), which was necessary due to our large sample size and the richness of the open-ended responses. Considering that this is a very time-consuming and task-intensive process, we followed a general inductive approach that allowed us to derive our findings from the most common and dominant themes in the raw data (29, 30) and although the analytical strategy of this approach is not very powerful (30), it’s simple and systematic approach is suitable for the purpose of this study as well as for the quality and quantity of the data. Two researchers independently coded 10 transcripts at a time and met to compute a Kappa statistic and discuss discrepancies in coding. This process was repeated until consensus was reached (Kappa statistic >0.70). The steps of this study were showed in Table 1.

**Table 1:** Steps of qualitative data analysis.

Steps	Content
Step 1	Immersive reading, where two researchers independently read all the data. Although the sample size is large, there is only one open-ended question and most participants’ responses are concise in length, which increases the ease of managing the text. Each researcher writes notes while reading the data to discover more codes.
Step 2	Both researchers explained the coding, and ensured the consistency of the coding scheme through discussion.
Step 3	The third researcher read all the data and assigned the responses to relevant codes. If necessary, additional codes were added after discussion. The other two researchers reviewed the consistency and completeness of the coding.
Step 4	All three researchers read the data and classified meaningful correlations and linkages between different codes.
Step 5	All researchers finalized the names and definitions of categories and codes and counted frequencies, summarized themes and identified exemplar quotes. Any disagreements were resolved through discussion and negotiation.

### Ethical consideration

The study was approved by Soochow University (with the codes SUDA20240626H01) and was conducted in accordance with the Declaration of Helsinki. Informed consent was embedded at the beginning of the online questionnaire and participants were prompted to withdraw at any time. Participants completed and submitted the electronic questionnaire indicating that their informed consent had been obtained.

## Results

### Participants

The study included 541 cancer patients, 267 of whom were female (49.4%). The participants’ mean age was 53.37 years (SD = 14.38). More than half of the participants (81.0%) were married, and 87.8% participants at early to mid-stage cancer stage. Table 2 shows the detailed characteristics.

**Table 2:** Sociodemographic information of participants (*n* = 541).

Variables	*n* (%)
Age	
<30	34 (6.3)
30 ~ 60	317 (58.6)
>60	190 (35.1)
Gender	
Male	274 (50.6)
Female	267 (49.4)
Marital status	
Married	438 (81.0)
Single	35 (6.5)
Divorced or widowed	68 (12.5)
Ethnicity	
Han	431 (79.7)
Others	110 (20.3)
Religion	
Yes	39 (7.2)
No	502 (92.8)
Education status	
Primary and below	60 (11.1)
Junior high school	187 (34.6)
Senior high school	226 (41.8)
Undergraduate and above	68 (12.6)
Occupational status	
Employed	238 (44.0)
Retirement	207 (38.3)
Others	96 (17.7)
Residence	
Urban	308 (56.9)
Rural	233 (43.1)
Average family income per month (yuan)	
<2000	74 (13.7)
2000 ~ 5,000	225 (41.6)
5,001 ~ 10,000	211 (39.0)
>10,000	31 (5.7)
Type of medical insurance	
New rural cooperative medical system	299 (55.3)
Urban employee basic medical insurance	207 (38.3)
Self-funded	7 (1.3)
Publicly funded	18 (3.3)
Others	10 (1.8)
Primary caregivers	
Spouses	275 (50.8)
Children	189 (34.9)
Parents	30 (5.5)
Others	47 (8.7)
Duration of illness (year)	
<1	128 (23.7)
1 ~ 5	332 (61.4)
>5	81 (15.0)
Cancer stage	
I	197 (36.4)
II or III	278 (51.4)
IV	66 (12.2)
Level of pain	
Mild	291 (53.8)
Moderate	213 (39.4)
Severe	37 (6.8)
Level of anxiety	
None	139 (25.7)
Mild	270 (49.9)
Moderate	119 (22.0)
Severe	13 (2.4)
Level of depression	
None	159 (29.4)
Mild	256 (47.3)
Moderate	113 (20.9)
Severe	13 (2.4)
Level of disease knowledge	
Completely unknown	24 (4.4)
Unknown	79 (14.6)
Neutral	160 (29.6)
Known	222 (41.0)
Completely known	56 (10.4)
Level of palliative care knowledge	
Completely unknown	263 (48.6)
Unknown	203 (37.5)
Neutral	55 (10.2)
Known	19 (3.5)
Completely known	1 (0.2)
Have you been received palliative care-related education or training?	
Yes	26 (4.8)
No	515 (95.2)

### Scores of palliative care attitudes among cancer patients

Participants had a total palliative care attitudes score of (27.38 ± 3.59). The scores for each item can be seen in Supplementary Table 1 and Supplementary Figure 1, with Item 5 “Do you think a palliative care consultation would help with feelings of sadness and depression?” rating as the highest, whereas Item 3 “How stressful would you find discussing emotions, like feeling sad, scared, or angry?” had the lowest.

### Influencing factors of palliative care attitudes

There were statistically significant differences in palliative care attitudes by cancer patients’ ethnicity, religion, education status, occupational status, residence, average family income per month, type of medical insurance, primary caregivers, cancer stage, anxiety, depression, disease knowledge, palliative care knowledge, and palliative care-related education or training experiences (*p* < 0.05) in single-factor analysis (Supplementary Table 2). Considering the variables that were statistically different in the single-factor analysis as independent variables and the total palliative care attitude score as the dependent variable, the results of the multiple linear regression analysis showed that religion, average family income per month, primary caregiver, and palliative care knowledge were the main influences on cancer patients’ palliative care attitudes (*p* < 0.05) (Supplementary Table 3).

### Profile model selection

**Figure 1:**
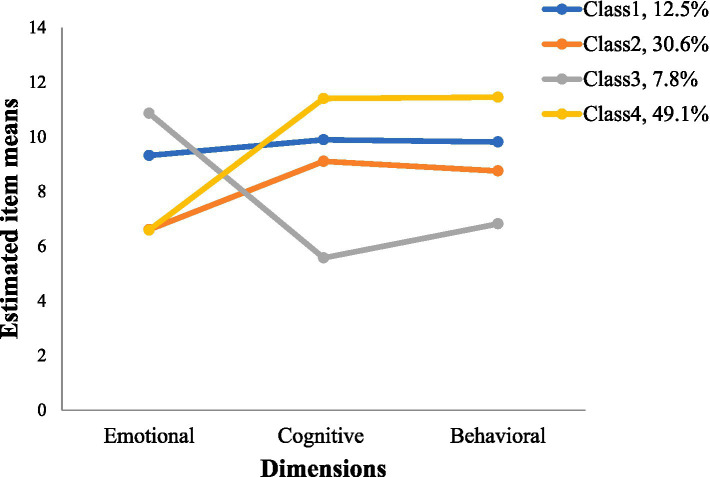
The latent profiles of the palliative care attitudes dimensions.

Using the Palliative Care Attitudes Scale total score as the indicator, the model was fitted to possible profiles 1 through 5 in the present study. According to the *p*-values for LMR and BLRT, the five-profile model was excluded. The AIC, BIC, and ABIC were decreasing in entropy, indicating that the profile model performs better as the number of profiles increases. The entropy value is higher in four-profile compared to three-profile and the probability of each category is greater than 5% for the four-profile model, the four-profile model was found to be the best suited model with high classification certainty, taking into account the previously mentioned data (Table 3 and Figure 1).

**Table 3:** Model fitting indexes for LPA in palliative care attitudes (*n* = 541).

Model	AIC	BIC	ABIC	LMR (*p*)	BLRT (*p*)	Entropy	Probabilities of classes
1	6914.610	6940.370	6921.324	–	–	–	100%
2	6580.539	6623.474	6591.730	0.000	0.000	0.932	89.2%/10.8%
3	6491.543	6551.651	6507.210	0.000	0.000	0.716	44.3%/8.6%/47.1%
4	6442.122	6519.404	6462.265	0.045	0.000	0.734	12.5%/30.6%/7.8%/49.1%
5	6411.851	6506.306	6436.470	0.178	0.000	0.777	7.4%/6.0%/38.4%/37.7%/10.5%

AIC, Akaike Information Criterion; BIC, Bayesian Information Criterion; aBIC, Sample-size-adjusted Bayesian Information Criterion; LMR, Lo–Mendell–Rubin Adjusted Likelihood Ratio Test; BLRT, Bootstrap Likelihood Ratio Test.

Class 4 presented the highest palliative care cognitive and behavioral scores but lowest emotional scores (named the ‘low-emotional, high-cognitive & behavioral group’). Class 3 showed low level of cognitive and behavioral scores but highest emotional scores (named the ‘high-emotional, low cognitive & behavioral group’). Class 2 had the lowest emotional scores (named the ‘low-emotional group’). Class 1 had the overall more even and not too high scores (named the ‘balanced negative group’).

Multivariate logistic regression result was showed in Table 4. The profiles of palliative care attitudes were used as the dependent variable, with the “low emotional, high-cognitive & behavioral group” as the reference. Sociodemographic information and disease-related information served as independent factors in the analysis. The findings revealed a statistically significant effect of education status, occupational status, primary caregivers, type of insurance, cancer stage, anxiety, and level of palliative care knowledge on the different profiles of palliative care attitudes (*p* < 0.05).

**Table 4:** Results of multivariate logistic regression analysis (*n* = 541).

Variable	Balanced negative group (ref. low-emotional, high-cognitive & behavioral group)						Low-emotional group (ref. low-emotional, high-cognitive & behavioral group)						High-emotional, low cognitive & behavioral group (ref. low-emotional, high-cognitive & behavioral group)					
	*β*	SE	OR	95%CI		*p*	*β*	SE	OR	95%CI		*p*	*β*	SE	OR	95%CI		*p*
				Lower	Upper					Lower	Upper					Lower	Upper	
Age (ref. > 60)																		
<30	−0.678	1.185	0.508	0.050	5.180	0.567	0.229	0.775	1.258	0.276	5.741	0.767	−1.576	2.675	0.207	0.001	39.139	0.556
30 ~ 60	−0.373	0.542	0.688	0.238	1.992	0.491	0.148	0.354	1.159	0.579	2.320	0.676	−0.696	0.919	0.498	0.082	3.019	0.449
Gender (ref. female)																		
Male	−0.007	0.357	0.993	0.493	1.998	0.984	0.161	0.227	1.175	0.752	1.835	0.479	−0.284	0.812	0.753	0.153	3.698	0.727
Marital status (ref. divorced or widowed)																		
Single	0.147	0.584	1.159	0.369	3.640	0.801	0.177	0.381	1.194	0.566	2.517	0.641	0.811	1.194	2.250	0.217	23.350	0.497
Married	1.593	1.754	4.920	0.158	15.124	0.364	−1.235	1.422	0.291	0.018	4.727	0.385	4.358	3.176	78.074	0.155	3.918	0.170
Ethnicity (ref. others)																		
Han	−0.068	0.503	0.934	0.348	2.506	0.893	−0.361	0.284	0.697	0.400	1.216	0.204	1.026	1.341	2.789	0.201	3.635	0.444
Religion (ref. no)																		
Yes	−0.056	0.780	0.945	0.205	4.362	0.942	0.537	0.424	1.711	0.746	3.924	0.205	2.159	1.441	8.659	0.514	1.934	0.134
Education status (ref. undergraduate and above)																		
Primary and below	2.735	1.206	15.409	1.449	163.819	0.023	0.376	0.643	1.456	0.413	5.135	0.559	−4.006	2.270	0.018	0.000	1.556	0.078
Junior high school	1.423	1.088	4.150	0.492	34.980	0.191	0.064	0.507	1.066	0.395	2.881	0.899	−4.138	1.886	0.016	0.000	0.644	0.028
Senior high school	1.659	1.007	5.253	0.729	37.840	0.100	−0.340	0.439	0.711	0.301	1.682	0.438	−4.546	1.899	0.011	0.000	0.438	0.017
Occupational status (ref. others)																		
Employed	−0.826	0.665	0.438	0.119	1.613	0.215	−0.365	0.440	0.694	0.293	1.644	0.407	−0.107	1.309	0.899	0.069	11.739	0.935
Retirement	−1.387	0.590	0.250	0.079	0.795	0.019	−0.848	0.416	0.428	0.190	0.968	0.042	−0.991	1.079	0.371	0.045	3.078	0.359
Residence (ref. rural)																		
Urban	−1.776	0.935	0.169	0.027	1.057	0.057	−1.303	0.746	0.272	0.063	1.172	0.081	−1.982	1.975	0.261	0.151	3.192	0.315
Average family income per month (ref. >10000)																		
<2000	1.256	1.550	3.512	0.168	73.284	0.418	1.481	0.806	4.398	0.906	21.361	0.066	2.263	1.759	3.207	0.452	2.382	0.987
2000 ~ 5,000	1.223	1.388	3.398	0.224	51.580	0.378	0.735	0.683	2.085	0.547	7.946	0.282	1.705	1.558	3.046	0.767	2.523	0.990
5,001 ~ 10,000	1.254	1.312	3.505	0.268	45.898	0.339	0.753	0.621	2.123	0.628	7.175	0.225	1.065	1.457	2.914	0.825	3.947	0.991
Type of medical insurance (ref. others)																		
New rural cooperative medical system	−1.808	1.540	0.164	0.008	3.357	0.240	−0.653	1.034	0.521	0.069	3.948	0.528	−4.205	2.428	0.015	0.000	1.739	0.083
Urban employee basic medical insurance	−3.201	1.774	0.041	0.001	1.317	0.071	−1.996	1.212	0.136	0.013	1.462	0.100	−0.864	2.866	0.421	0.002	11.885	0.763
Self-funded	−3.113	2.086	0.044	0.001	2.650	0.136	−2.308	1.537	0.099	0.005	2.022	0.133	−6.716	3.750	0.499	0.537	2.276	0.996
Publicly funded	−2.657	2.106	0.070	0.001	4.354	0.207	−3.239	1.460	0.029	0.002	0.686	0.027	−8.184	4.734	0.918	0.345	3.291	0.994
Primary caregivers (ref. others)																		
Spouses	−3.552	1.294	0.029	0.002	0.362	0.006	−1.859	1.243	0.156	0.014	1.782	0.135	−7.891	1.784	0.002	0.134	0.212	0.000
Children	−2.990	1.303	0.050	0.004	0.647	0.022	−1.746	1.255	0.174	0.015	2.042	0.164	−7.167	1.714	0.005	0.182	0.322	0.000
Parents	−4.078	2.047	0.017	0.007	0.936	0.046	−0.062	1.756	0.940	0.030	29.357	0.972	−2.361	3.524	0.714	1.284	2.692	0.998
Duration of illness (year) (ref. >5)																		
<1	−0.474	0.655	0.622	0.172	2.248	0.469	−0.070	0.404	0.932	0.423	2.058	0.863	0.749	1.260	2.116	0.179	24.997	0.552
1 ~ 5	−0.348	0.578	0.706	0.227	2.195	0.366	−0.078	0.337	0.925	0.478	1.790	0.818	0.069	1.158	1.071	0.111	10.353	0.953
Cancer stage (ref. IV)																		
I	1.041	1.262	2.833	0.239	33.591	0.409	−0.363	0.582	0.695	0.222	2.178	0.533	−3.661	1.520	0.026	0.001	0.506	0.016
II or III	1.088	1.204	2.970	0.281	31.423	0.366	0.295	0.494	1.343	0.510	3.537	0.550	−2.661	1.387	0.070	0.005	1.059	0.055
Level of pain (ref. severe)																		
Mild	0.515	1.318	1.376	0.956	4.281	0.992	0.428	0.671	1.535	0.412	5.714	0.523	1.453	1.314	2.369	0.823	3.291	0.988
Moderate	0.468	0.927	1.103	0.784	3.930	0.992	0.168	0.600	1.183	0.365	3.835	0.779	1.006	1.015	1.494	0.374	2.010	0.988
Level of anxiety (ref. severe)																		
None	−0.454	0.981	0.820	0.110	2.439	0.995	−0.445	0.766	0.641	0.143	2.876	0.641	−5.719	2.667	0.003	1.745	0.618	0.032
Mild	−0.915	0.473	0.396	0.042	1.460	0.995	−1.023	0.721	0.360	0.088	1.478	0.360	−6.962	2.473	0.001	7.444	0.121	0.005
Moderate	−0.558	0.608	0.327	0.064	1.842	0.995	−0.828	0.712	0.437	0.108	1.765	0.437	−8.406	2.666	0.001	1.202	0.042	0.002
Level of depression (ref. severe)																		
None	−0.532	0.791	0.653	0.143	3.292	0.995	−0.168	0.737	0.845	0.199	3.580	0.819	−1.566	1.320	0.452	0.329	3.029	0.994
Mild	−0.828	0.573	0.533	0.104	2.483	0.995	−0.675	0.717	0.509	0.125	2.077	0.347	−1.627	1.217	0.511	0.483	2.956	0.994
Moderate	−0.701	0.604	0.547	0.128	2.735	0.995	−0.834	0.699	0.434	0.110	1.709	0.233	−1.201	1.290	0.735	0.204	2.402	0.995
Level of disease knowledge (ref. completely known)																		
Completely unknown	0.456	1.215	1.577	0.146	17.050	0.708	−1.120	0.777	0.326	0.071	1.495	0.149	−1.751	1.785	1.954	0.000	0.000	0.992
Unknown	0.323	0.992	1.381	0.198	9.654	0.745	0.183	0.557	1.200	0.403	3.576	0.743	−2.435	1.583	0.088	0.004	1.951	0.124
Neutral	0.268	0.923	1.307	0.214	7.954	0.772	−0.338	0.509	0.713	0.263	1.934	0.507	−2.759	1.627	0.063	0.003	1.538	0.090
Known	0.011	0.898	1.011	0.174	5.878	0.990	−0.052	0.470	1.053	0.419	2.643	0.912	−1.036	1.341	0.355	0.026	4.914	0.440
Level of palliative care knowledge (ref. completely known)																		
Completely unknown	1.633	1.433	4.581	0.681	3.934	0.000	16.644	0.874	3.493	1.313	3.631	0.000	5.267	1.744	2.815	0.012	1.593	0.999
Unknown	1.269	1.332	2.515	0.758	3.40	0.000	15.962	0.878	3.577	1.934	4.712	0.000	4.792	1.412	1.576	0.032	1.714	0.999
Neutral	1.522	1.436	3.333	0.096	2.157	0.000	16.407	0.886	4.795	2.137	7.608	0.000	−3.788	1.247	0.027	0.057	2.146	0.999
Known	1.003	0.842	2.012	0.372	2.372	0.000	16.115	0.000	4.818	3.618	9.618	0.000	−3.334	1.109	0.036	0.129	2.971	0.999
Have you been received palliative care-related education or training? (ref. no)																		
Yes	−0.189	1.273	0.828	0.068	10.044	0.882	−1.038	0.846	0.354	0.067	1.861	0.220	−1.111	1.028	0.752	0.073	8.034	0.994

### The qualitative analysis

**Figure 2:**
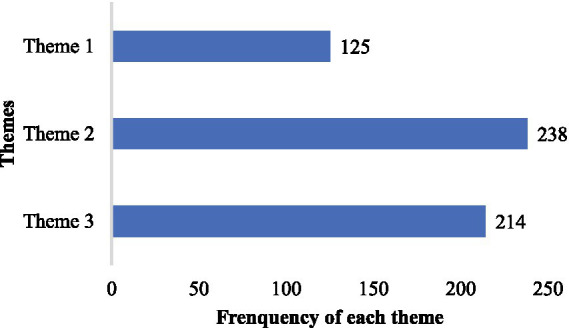
Frequency of each theme in palliative care perspective categories.

Cancer patients’ perspectives on palliative care were divided into three themes: “theme 1, expectations for the promotion of palliative care services (21.7%),” “theme 2, skepticism and refusal toward palliative care development (41.2%),” “theme 3, indifference to palliative care due to limited knowledge (37.1%).” Figure 2 shows the frequency of occurrence of each theme, the most common perspective is theme 2. We discuss each theme according to its order of frequency, with example statements from responses.

In qualitative analysis, the most common theme identified was “skepticism and refusal toward palliative care development.” Fifty-eight (10.7%) patients explicitly rejected palliative care. “I’ve been to many hospitals and tried numerous treatment plans; I will not believe in any new methods anymore,” “There is no palliative care available in township hospitals, which is inconvenient,” “The cost must be high, and I do not want to waste money anymore,” and “I’ve never heard of palliative care before, are you trying to experiment with my life?.” Additionally, 151 (27.9%) patients doubt palliative care, asking “What is palliative care, and can it really prolong my life?” “Is palliative care treatment or care?” “Can palliative care relieve my pain?” “As long as it can reduce my depression, I’m willing to try,” “Where can I consult about palliative care?” “Can palliative care consultation be done online?” and “Is palliative care euthanasia? I’m scared when I hear that word.”

The second frequently encountered theme is “indifference to palliative care due to limited knowledge.” In the final phase of the quantitative study, an open-ended question was posed, allowing participants to provide any text-based responses, including thoughts, suggestions, and queries. However, 140 (25.9%) participants answered only “none” and 62 (11.5%) participants with “I have no idea because I have never known palliative care before.” The remaining minority responded with “First time hearing about palliative care.”

Although Theme 3 implies a positive attitude toward palliative care, it has the lowest frequency. Of the 69 (12.7%) participants, a desire to engage in palliative care was expressed. Responses such as “I wish to experience palliative care” “I am interested and would like to seek palliative care consultation” and “This is my first encounter with palliative care, and I will arrange a consultation promptly” were common. 36 (6.7%) participants mentioned their desire to further understand palliative care and intention to pay attention to it. 15 (2.8%) participants expressed belief in palliative care, stating sentiments like “I hope palliative care becomes more widespread, so I can benefit from it even in rural hospitals” and “I heard palliative care before, it is a comprehensive treatment, and I hope it continues to improve.”

## Discussion

This study found that palliative care attitudes were poor among cancer patients and explained the causes of this phenomenon through both quantitative and qualitative research designs. As we know, for the first time in a quantitative study, LPA was used to identify the role of individual variability in contributing to different attitudinal classes toward palliative care, with the “low-emotional, high-cognitive & behavioral group” being most over-represented. Education status, occupational status, primary caregivers, type of insurance, cancer stage, anxiety, and level of palliative care knowledge were identified as the main influencing factor. In the qualitative study, content analysis of a large sample of responses identified three themes that may further our understanding of the sources of individual variability in palliative care attitudes. In conclusion, poor attitudes toward palliative care were found among Chinese cancer patients was founded, which require collaboration among health policymakers, hospital administrators, health professionals and social volunteers to improve public understanding and acceptance of palliative care.

In this study, 477 (86.1%) cancer patients were unaware of palliative care, among whom 263 (48.6%) had never heard of it, consistent with the previous study (16). Cancer patients in China demonstrated overall low scores in palliative care attitudes, indicating a lower acceptance due to insufficient knowledge. China, deeply influenced by traditional culture, historically adhered to Confucianism advocating “enjoy life, detest death,” leading to the “taboo” nature of discussing death for most publics (13, 31). The promotion of palliative care began officially in China in 2017; however, despite the government’s emphasis on providing palliative care services, resources remain scarce, with insufficient education and training hindering healthcare providers from promptly delivering palliative care to patients in need (32). Notably, one prominent finding revealed is that about half of cancer patients are completely unknown of palliative care, which may become a crucial influencing factor on attitudes, as a lack of actionable information tends to prompt individuals to make negative or avoidant decisions (33). Therefore, governmental efforts should mobilize healthcare professionals, community members, volunteers to intensify the dissemination of palliative care knowledge. Media platforms can be utilized to increase cancer patients’ awareness and knowledge of palliative care, thereby improving their attitudes and acceptance.

In the balanced negative group, the lower education level, the more positive palliative care attitudes, which is consistent with the United States (34). Conversely, in the high emotional, low cognitive & behavioral group, the higher the level of education, the more positive the palliative care attitudes were found. The reason for these differences may be that in the balanced negative group, the lower education level of the cancer patients implies weaker judgment and a greater likelihood of responding to a palliative care that tends to be accepting even if they do not know about it. In contrast, in the high emotional, low cognitive & behavioral group, better-educated patients were more likely to be knowledgeable and accept about palliative care.

Different caregivers also have varying effects on cancer patients’ palliative care attitudes. This study revealed that attitudes were more favorable when the caregivers were patient’s children compared to spouses and parents. This phenomenon may be attributed to the fact that younger children are more open-minded, have a better understanding of new policies, and exhibit a higher level of acceptance of palliative care as a new model of care. Thus, they may positively influence patients’ palliative care attitudes during the caregiving.

Patients in the high emotional, low cognitive & behavioral group with higher anxiety displayed worse palliative care attitudes. Anxiety, a common emotion in patients following a diagnosis, poses a serious threat to patient prognosis. Higher anxiety levels indicate worse emotional states and impaired emotional control, leading to a more negative attitude toward new concepts (35).

Through LPA analysis, this study found that influencing factors for diverse profiles of palliative care varied significantly, even yielding contradictory results. This suggests that healthcare providers should tailor assessment of palliative care attitudes for cancer patients and formulate intervention plans based on unique influencing factors. This approach aims to effectively enhance cancer patients’ attitudes toward palliative care and improve the quality of end-of-life care for patients.

The most common theme in the qualitative study was “skepticism and refusal toward palliative care development,” with 238 (44.0%) patients questioning and unbelieve the effectiveness of palliative care, and some participants even misunderstood palliative care as equal to euthanasia, and these misperceptions were mostly attributed to ignorance. Therefore, these findings highlight the importance of palliative care education. Additional, initiating early palliative care discussions can also help improve patients’ attitudes toward palliative care (19). However, it is reassuring that even if it is the first time, a small proportion expressed interest and hoped for its wider adoption in hospitals. It is evident that patients remain curious about palliative care, including its content, cost, format, and effectiveness, and express expectations for increased government investment in palliative care to ensure patient benefits. There is a need to develop seminars or workshops to help patients understand palliative care better.

### Implications for future researches

The findings of this study provide significant insights into cancer patients’ poor attitudes toward palliative care in China, emphasizing the need for further exploration and intervention. Future research could investigate additional psychosocial variables, such as cultural beliefs, religious affiliations, and social support systems, which may further elucidate the complexity of palliative care attitudes around worldwide. Additionally, future research should develop and evaluate targeted educational interventions, the focus of which can be tailored to the patient’s education level, occupational status, level of palliative knowledge, etc., to accurately raise public awareness. By doing so, researchers can develop evidence-based strategies to enhance public acceptance and ultimately improve the quality of end-of-life care.

### Limitations

There are several limitations. Firstly, there is a limited number of young and middle-aged cancer patients among the participants. Although the sample size is a strength, the uneven distribution of age groups may weaken the representativeness of the sample. Secondly, the textual responses were collected from qualitative questions in an online survey. Despite many responses being comprehensive, they still lack the depth that qualitative study can produce. We suggest further conducting rigorous qualitative research to deepen the study results. Lastly, we performed frequency calculations on the themes identified in the qualitative research results. Due to some responses being more elaborate, they were coded multiple times, which may lead to some responses being overrepresented in the themes.

## Conclusion

In conclusion, the multi-method design used in this study—quantitative comparisons followed by qualitative analyses—provides a more comprehensive view of cancer patients’ attitudes toward palliative care than studies that use only qualitative or quantitative methods. The findings highlight the urgent need for palliative care education and counseling for cancer patients. We recommend that, in addition to raising awareness of the aims and importance of palliative care in society, active efforts should be made to change cancer patients’ misconceptions in order to increase acceptance of palliative care. As family caregivers largely assume the role of surrogate decision-makers for cancer patients, health care providers must also consider training family caregivers in palliative care-related knowledge and skills and, where appropriate, actively promote patient-and family-caregiver-centered models of specialist palliative care practice. While gaps in knowledge can be addressed through a variety of ways, the influence of underlying values is more likely to determine cancer patients’ attitudes toward palliative care, thus, future research needs to focus on relevant influences in order to develop targeted strategies to promote early referral to palliative care.

## Data Availability

The original contributions presented in the study are included in the article/Supplementary material, further inquiries can be directed to the corresponding author.
