# Corrigendum: Attitudes toward palliative care among cancer patients: a multi-method study

**DOI:** 10.3389/fpubh.2025.1598820

**Published:** 2025-04-08

**Authors:** Meiying Zhang, Yuxia Zhao, Yifu Lu, Mengyun Peng

**Affiliations:** ^1^Department of Diagnostic Imaging, National Cancer Center/National Clinical Research Center for Cancer/Cancer Hospital, Chinese Academy of Medical Sciences and Peking Union Medical College, Beijing, China; ^2^Shantou University Medical College, Shantou University, Shantou, China; ^3^School of Nursing, Suzhou Medical College, Soochow University, Suzhou, China

**Keywords:** palliative care, attitudes, cancer, quantitative study, qualitative study

In the published article, there was an error in affiliation(s) 1 and 2. Instead of “1 Department of Outpatient, Cancer Hospital Chinese Academy of Medical Sciences, Beijing, China; 2 Department of Diagnostic Imaging, Cancer Hospital Chinese Academy of Medical Sciences, Beijing, China,” it should be “1 Department of Diagnostic Imaging, National Cancer Center/National Clinical Research Center for Cancer/Cancer Hospital, Chinese Academy of Medical Sciences and Peking Union Medical College, Beijing, China.”

The authors apologize for this error and state that this does not change the scientific conclusions of the article in any way. The original article has been updated.

