# Supplementary material for: Attitudes toward palliative care among cancer patients: a multi-method study
Source: Front Public Health. 2025 Mar 5;13:1511697. doi: 10.3389/fpubh.2025.1511697 (PMC11920124; doi:10.3389/fpubh.2025.1511697)
Supplement: Supplementary file 1 [file Table_1.docx]

**Supplementary Table 1. Mean score of each item.**

| Items | Min | Max | Mean | SD | Rate |
| --- | --- | --- | --- | --- | --- |
| 1.How stressful would you find it to be overall? | 1 | 5 | 2.85 | 0.835 | 7 |
| 2.How stressful would you find discussing severe physical symptoms or side effects (e.g., painful bone tumor, severe nausea, problems swallowing food)? | 1 | 5 | 2.35 | 0.685 | 8 |
| 3.How stressful would you find discussing emotions, like feeling sad, scared, or angry? | 1 | 5 | 2.07 | 0.599 | 9 |
| 4.Do you think a Palliative Care Consultation would help with physical quality of life? | 1 | 5 | 3.53 | 0.790 | 3 |
| 5.Do you think a Palliative Care Consultation would help with feelings of sadness and depression? | 1 | 5 | 3.63 | 0.967 | 1 |
| 6.Do you think a Palliative Care Consultation would help prolong life? | 1 | 5 | 2.89 | 1.078 | 6 |
| 7.Would you be willing to attend the Consultation? | 1 | 5 | 3.61 | 0.676 | 2 |
| 8.Would you try to schedule it as soon as possible? | 1 | 5 | 3.40 | 0.852 | 4 |
| 9.Would you be willing to attend on a monthly basis for several months if requested? | 1 | 5 | 3.05 | 1.042 | 5 |

**S****upplementary Table 2. Sociaodemographic differences in palliative care attitudes among cancer patients (*n* = 541).**

| Variables | *n* (%) | Palliative care attitudes  (M ± SD) | *t*/*F* | *p* |
| --- | --- | --- | --- | --- |
| Age |  |  | 1.710 | 0.182 |
| ＜30 | 34(6.3) | 28.18 ± 3.195 |  |  |
| 30~60 | 317(58.6) | 27.48 ± 3.469 |  |  |
| ＞60 | 190(35.1) | 27.06 ± 3.833 |  |  |
| Gender |  |  | 0.483 | 0.629 |
| Male | 274(50.6) | 27.45 ± 3.738 |  |  |
| Female | 267(49.4) | 27.30 ± 3.438 |  |  |
| Marital status |  |  | 1.963 | 0.141 |
| Married | 438(81.0) | 27.40 ± 3.427 |  |  |
| Single | 35(6.5) | 28.26 ± 3.681 |  |  |
| Divorced or widowed | 68(12.5) | 26.79 ± 4.437 |  |  |
| Ethnicity |  |  | 2.140 | 0.033 |
| Han | 431(79.7) | 27.55 ± 3.448 |  |  |
| Others | 110(20.3) | 26.73 ± 4.054 |  |  |
| Religion |  |  | -3.164 | 0.002 |
| Yes | 39(7.2) | 25.62 ± 3.794 |  |  |
| No | 502(92.8) | 27.51 ± 3.543 |  |  |
| Education status |  |  | 8.885 | ＜0.001 |
| Primary and below | 60(11.1) | 26.17 ± 3.892 |  |  |
| Junior high school | 187(34.6) | 26.63 ± 3.760 |  |  |
| Senior high school | 226(41.8) | 28.01 ± 3.117 |  |  |
| Undergraduate and above | 68(12.6) | 28.32 ± 3.711 |  |  |
| Occupational status |  |  | 13.998 | ＜0.001 |
| Employed | 238(44.0) | 27.89 ± 3.292 |  |  |
| Retirement | 207(38.3) | 27.58 ± 3.539 |  |  |
| Others | 96(17.7) | 25.69 ± 3.932 |  |  |
| Residence |  |  | -3.462 | ＜0.001 |
| Urban | 308(56.9) | 26.92 ± 3.692 |  |  |
| Rural | 233(43.1) | 27.00 ± 3.364 |  |  |
| Average family income per month (yuan) |  |  | 23.625 | ＜0.001 |
| ＜2000 | 74(13.7) | 24.68 ± 3.990 |  |  |
| 2000~5000 | 225(41.6) | 27.29 ± 3.492 |  |  |
| 5001~10000 | 211(39.0) | 28.09 ± 2.999 |  |  |
| ＞10000 | 31(5.7) | 29.68 ± 3.497 |  |  |
| Type of medical insurance |  |  | 4.126 | 0.003 |
| New rural cooperative medical system | 299(55.3) | 26.90 ± 3.653 |  |  |
| Urban employee basic medical insurance | 207(38.3) | 27.88 ± 3.371 |  |  |
| Self-funded | 7(1.3) | 27.19 ± 3.946 |  |  |
| Publicly funded | 18(3.3) | 29.56 ± 3.240 |  |  |
| Others | 10(1.8) | 27.50 ± 4.301 |  |  |
| Primary caregivers |  |  | 16.913 | ＜0.001 |
| Spouses | 275(50.8) | 28.19 ± 3.229 |  |  |
| Children | 189(34.9) | 26.67 ± 3.794 |  |  |
| Parents | 30(5.5) | 28.37 ± 4.222 |  |  |
| Others | 47(8.7) | 24.87 ± 2.473 |  |  |
| Duration of illness(year) |  |  | 2.350 | 0.096 |
| ＜1 | 128(23.7) | 26.91 ± 3.736 |  |  |
| 1~5 | 332(61.4) | 27.64 ± 3.583 |  |  |
| ＞5 | 81(15.0) | 27.05 ± 3.312 |  |  |
| Cancer stage |  |  | 5.308 | 0.005 |
| Ⅰ | 197(36.4) | 27.97 ± 3.355 |  |  |
| Ⅱ/Ⅲ | 278(51.4) | 27.18 ± 3.565 |  |  |
| Ⅳ | 66(12.2) | 26.47 ± 4.115 |  |  |
| Level of pain |  |  | 0.734 | 0.480 |
| Mild | 291(53.8) | 27.54 ± 3.370 |  |  |
| Moderate | 213(39.4) | 27.23 ± 3.904 |  |  |
| Severe | 37(6.8) | 26.97 ± 3.590 |  |  |
| Level of anxiety |  |  | 3.170 | 0.024 |
| None | 139(25.7) |  |  |  |
| Mild | 270(49.9) | 26.76 ± 3.417 |  |  |
| Moderate | 119(22.0) | 27.84 ± 3.509 |  |  |
| Severe | 13(2.4) | 27.09 ± 3.925 |  |  |
| Level of depression |  |  | 3.450 | 0.016 |
| None | 159(29.4) |  |  |  |
| Mild | 256(47.3) | 26.89 ± 3.188 |  |  |
| Moderate | 113(20.9) | 27.76 ± 3.527 |  |  |
| Severe | 13(2.4) | 27.45 ± 4.036 |  |  |
| level of disease knowledge |  |  | 3.375 | 0.010 |
| Completely unknown | 24(4.4) | 27.08 ± 3.562 |  |  |
| Unknown | 79(14.6) | 26.11 ± 4.224 |  |  |
| Neutral | 160(29.6) | 27.88 ± 3.340 |  |  |
| Known | 222(41.0) | 27.46 ± 3.300 |  |  |
| Completely known | 56(10.4) | 27.55 ± 4.103 |  |  |
| Level of palliative care knowledge |  |  |  |  |
| Completely unknown | 263(48.6) | 26.78 ± 3.815 | 8.413 | ＜0.001 |
| Unknown | 203(37.5) | 27.60 ± 3.175 |  |  |
| Neutral | 55(10.2) | 28.20 ± 2.984 |  |  |
| Known | 19(3.5) | 30.58 ± 3.485 |  |  |
| Completely known | 1(0.2) | 36.00 ± 0.000 |  |  |
| Have you been received palliative care-related education or training? |  |  | 5.528 | ＜0.001 |
| Yes | 26(4.8) | 31.08 ± 3.452 |  |  |
| No | 515(95.2) | 27.19 ± 3.498 |  |  |

**Supplementary Table 3.** **Multiple regression analysis of palliative care attitudes.**

| Variables | B | SE | β | *t* | *p* | 95%CI | |
| --- | --- | --- | --- | --- | --- | --- | --- |
|  |  |  |  |  |  | Lower | Upper |
| Ethnicity | -0.546 | 0.377 | -0.061 | -1.447 | 0.148 | -1.286 | 0.195 |
| Religion | 1.652 | 0.577 | 0.119 | 2.860 | 0.004 | 0.517 | 2.786 |
| Education status | 0.027 | 0.238 | 0.006 | 0.115 | 0.908 | -0.439 | 0.494 |
| Occupational status | 0.123 | 0.256 | 0.025 | 0.482 | 0.630 | -0.380 | 0.626 |
| Residence | -0.234 | 0.393 | -0.032 | -0.595 | 0.552 | -1.005 | 0.538 |
| Average family income per month | 1.017 | 0.270 | 0.223 | 3.764 | ＜0.001 | 0.486 | 1.547 |
| Type of medical insurance | 0.151 | 0.213 | 0.035 | 0.709 | 0.479 | -0.267 | 0.569 |
| primary caregivers | -0.769 | 0.189 | -0.196 | -4.074 | ＜0.001 | -1.140 | -0.398 |
| Cancer stage | 0.177 | 0.242 | 0.030 | 0.730 | 0.466 | -0.299 | 0.652 |
| Anxiety | 0.040 | 0.231 | 0.008 | 0.172 | 0.863 | -0.414 | 0.493 |
| Depression | -0.176 | 0.230 | -0.038 | -0.763 | 0.446 | -0.628 | 0.277 |
| Disease knowledge | -0.151 | 0.159 | -0.042 | -0.951 | 0.342 | -0.464 | 0.161 |
| Palliative care knowledge | -2.475 | 0.757 | -0.148 | -3.270 | 0.001 | -3.962 | -0.988 |
| Palliative care-related education or training experiences | 0.319 | 0.208 | 0.072 | 1.535 | 0.125 | -0.089 | 0.726 |
