# Supplementary material for: Attitudes toward palliative care among cancer patients: a multi-method study
Source: Front Public Health. 2025 Mar 5;13:1511697. doi: 10.3389/fpubh.2025.1511697 (PMC11920124; doi:10.3389/fpubh.2025.1511697)
Supplement: Supplementary file 2 [file Image_1.pdf]

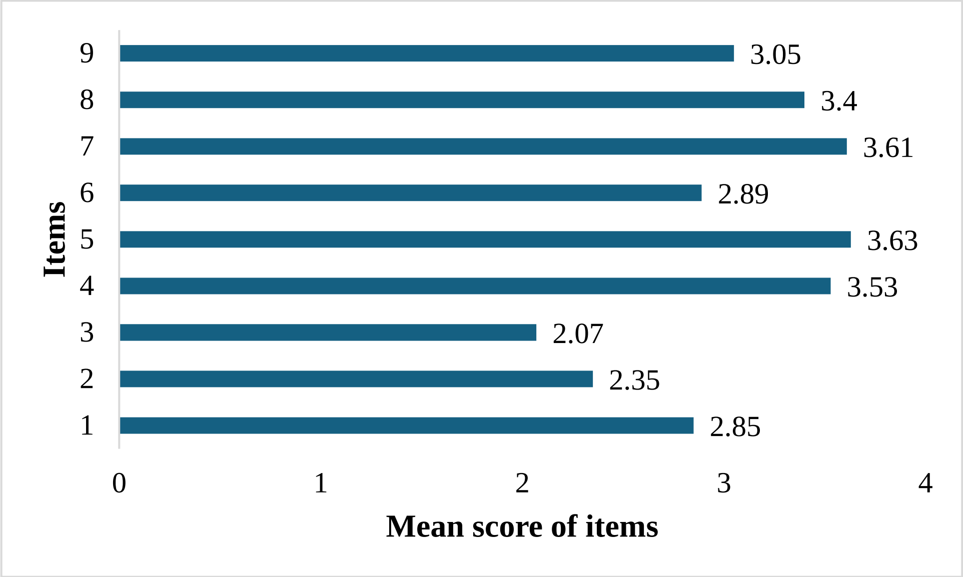

Supplementary Figure 1. Bar graph of mean scores for each item.

Note.

1. How stressful would you find it to be overall?
2. How stressful would you find discussing severe physical symptoms or side effects (e.g., painful bone tumor, severe nausea, problems swallowing food)?
3. How stressful would you find discussing emotions, like feeling sad, scared, or angry?
4. Do you think a Palliative Care Consultation would help with physical quality of life?
5. Do you think a Palliative Care Consultation would help with feelings of sadness and depression?
6. Do you think a Palliative Care Consultation would help prolong life?
7. Would you be willing to attend the Consultation?
8. Would you try to schedule it as soon as possible?
9. Would you be willing to attend on a monthly basis for several months if requested?
